# The effect of sleep duration in clinical features and impact of migraine: Result from a population-based study

**DOI:** 10.1186/1129-2377-14-S1-P6

**Published:** 2013-02-21

**Authors:** BK Kim, JM Kim, KS Lee, MK Chu

**Affiliations:** 1 Department of Neurology, Eulji University School of Medicine, Korea, Republic of; 2 Department of Neurology, Chungnam National University, College of Medicine, Korea, Republic of; 3 Department of Neurology, Seoul St.Mary's Hospital, The Catholic University of Korea, Korea, Republic of; 4 Department of Neurology, Hallym University College of Medicine, Korea, Republic of

## Background

Although sleep disturbances are a common complaint in migraine patients, the role of sleep habits such sleep duration in clinical features and impact has been poorly analyzed.

## Objective

To assess the influence of sleep duration on clinical features and impact of migraine.

## Methods

We selected a stratified random population sample of Koreans over age 19 and evaluated them with a 60-item semi-structured interview designed to identify headache type using ICHD-2 criteria and sleep status such as sleep duration and sleep onset time. We also included items for demographics and HIT-6.

## Results

Of 2,836 all participants, 152 were diagnosed as having migraine. The mean sleep duration similar between migraineurs (7.1±1.5 hours) and non-migraine controls (7.1±1.3 hours). Among migraineurs, 15 (9.9%) participants slept ¡Â5 hours, 83 (54.6%) slept 5-7 hours, 44 (28.9%) slept 7-9 hours, and 10 (6.6%) slept >9 hours in weekdays. Migraineurs with sleep duration of ≤5 hours reported higher migraine attack frequency (9.8±11.3 attacks per month) comparing to a sleep duration of >5 hours (3.8±6.3 attacks per month, p=0.001). Migraineurs with ≤5 hours sleep duration showed a tendency of increased HIT-6 score (59.7±9.9) comparing to sleep duration of 7-9 hours (53.1±5.8, p=0.088). Unilateral pain was more prevalent among migraineurs with sleep duration of >5 hours comparing to sleep duration of ≤5 hours. Headache severity, pulsating quality, aggravation by movement, nausea, vomiting, photophobia and phonophobia was not significant according to sleep duration.

## Conclusions

High attack frequency is associated with sleep duration of ≤5 hours among migraineurs.
